# NVX-CoV2373 induces humoral and cellular immune responses that are functionally comparable to vector and mRNA-based vaccines

**DOI:** 10.3389/fimmu.2024.1359475

**Published:** 2024-03-18

**Authors:** Franz Mai, Marcel Kordt, Wendy Bergmann-Ewert, Emil C. Reisinger, Brigitte Müller-Hilke

**Affiliations:** ^1^ Institute of Immunology, Rostock University Medical Center, Rostock, Germany; ^2^ Core Facility for Cell Sorting and Cell Analysis, Rostock University Medical Center, Rostock, Germany; ^3^ Division of Tropical Medicine and Infectious Diseases, Center of Internal Medicine II, Rostock University Medical Center, Rostock, Germany

**Keywords:** BNT162b2, AZD1222, NVX-CoV2373, homologous and heterologous vaccination, SARS-CoV-2, antibody, T-cell, vaccine

## Abstract

**Background:**

After licensing of the protein-based vaccine NVX-CoV2373, three technically different vaccines against the SARS-CoV-2 became available for application to the human population - and for comparison of efficacies.

**Methods:**

We here recruited 42 study participants who had obtained one initial dose of NVX-CoV2373 and analyzed their immune responses in contrast to 37 study participants who had obtained either the vector vaccine AZD1222 or the mRNA vaccine BNT162b2 a year earlier. 32 participants also donated blood before first vaccination to serve as a vaccine-naive control. In detail, we investigated and quantified at day 21 and approximately six months after primary immunization the amounts of vaccine-specific antibodies produced, their neutralization capacity, their quality in terms of binding different epitopes and their efficiency in inducing various isotypes. Cellular immunity and intracellular cytokine production following *in vitro* re-stimulation with BNT162b2 vaccine was analyzed via ELISpot or via flow cytometry.

**Results:**

Our results show that even though vaccination including the mRNA vaccine yielded best results in almost any aspect of antibody levels and binding efficiency, the neutralization capacities against the wild-type Wuhan strain and the Omicron BA.1 variant early and at six months were comparable among all three vaccination groups. As for the T cells, we observed a prevailing CD8 response at three weeks which turned into a predominant CD4 memory at six months which has not yet been observed for AZD1222 and BNT162b2. While additional infection with SARS-CoV-2 resulted in a boost for the humoral response, T cell memory appeared rather unaffected.

**Conclusion:**

Whether any of these differences translate into real world protection from infection, mitigation of severe disease courses and prevention of long/post COVID will need to be investigated in the future.

## 1 Introduction

Following the outbreak of SARS-CoV-2 in December 2019 (1) and its classification as another human pathogenic species (2), the development of vaccines to combat Corona virus disease 2019 (COVID-19) commenced at record speed. Vaccines produced by using a novel mRNA technology, e.g. BNT162b2 from BioNTech and mRNA-1273 from Moderna, received their first approvals in December 2020 and January 2021, respectively. Likewise, AstraZeneca’s vector-based vaccine AZD1222 was approved in January 2021. All three vaccines showed high efficacy in preventing severe disease courses (3–5) and were also shown to mitigate the risk of long-term manifestation of COVID-19 (6, 7). We and others could show in head-to-head comparisons that homologous and heterologous use of mRNA vaccines resulted in a significantly stronger humoral immune response and that was true after two (8–11) and also after three doses of vaccine (12–15). In contrast, results for T cell memories were not as clear cut with some descriptions of mRNA vaccines being superior to the homologous vector regimen (16–18), while others published comparable results for both vaccines (9, 10, 19, 20). In December 2021, the protein-based vaccine NVX-CoV2373 (Nuvaxovid) developed by Novavax was licensed after also confirming high efficacy (21, 22). By now the scientific community is in the unprecedented situation that three completely different vaccination regimen can be scrutinized via real world data, large numbers and longitudinal studies for the induction of humoral and cellular immune responses, the effect of boosting, longevity of the immune memory, as well as possible adverse events. We here set out to follow up on voluntary study participants who obtained NVX-CoV2373 and analyzed their immune responses at three weeks and six months after their first vaccination in order to compare them with already established vaccination regimes. We thereby focused not only on the amounts of antibodies produced and their capacity to neutralize, but also on the vaccine`s ability to elicit various antibody isotypes. Moreover, we were curious how natural infection with SARS-CoV-2 impacted on the existing spike-specific humoral and cellular immune responses.

## 2 Material and methods

### 2.1 Blood samples

Human blood samples were collected via venipuncture at 21 days and approximately six month after primary immunization. Blood samples from the control group were collected before first vaccination. EDTA blood was centrifuged at 1500× g for 10 min to obtain plasma and untreated blood was centrifuged at 2000x g for 10 min to obtain serum. Both were frozen at −80°C for subsequent analyses of IgG antibodies towards spike receptor binding domain (RBD) and against nucleocapsid protein, for surrogate virus neutralization test (sVNT), and for binding of the spike protein expressing human T-cell line Jurkat clone E6-1. Peripheral blood mononuclear cells (PBMCs) were isolated by density gradient centrifugation (Ficoll‐PaqueTM PLUS, Cytiva, Marlborough, MA, USA) and stored in heat‐inactivated fetal calf serum (FCS, Thermo Fisher, Waltham, MA, USA) containing 10% dimethyl sulfoxide (Sigma‐Aldrich, St. Louis, MO, USA) at −80°C for later use in Interferon Gamma assay (ELISpot) and *in vitro* re-stimulation via flow cytometry.

### 2.2 Measurement of IgG, IgM and IgA antibodies against SARS‐CoV‐2 via ELISA

Enzyme-linked immunosorbent assay (ELISA) (Anti-SARS-CoV-2 ELISA (IgG/IgM/IgA), EUROIMMUN Medizinische Labordiagnostika AG, Lübeck, Germany) targeting the S1 domain of the spike protein was performed according to the manufacturer’s instructions. In short, plasma samples were incubated with the coated microtiter plate for 60 minutes at 37°C. Subsequently, the enzyme conjugate was incubated for 30 minutes at 37°C, chromogen substrate was added, and last incubation for 30 minutes at room temperature followed by addition of the stop solution. Photometric measurements were taken at 450nm using InfiniteM200 (Tecan, Männeheim, Switzerland). Optical densities (OD) of samples divided by the OD of positive controls resulted in arbitrary units.

### 2.3 Measurement of IgG antibodies against SARS‐CoV‐2 Nucleocapsid and RBD of SARS‐CoV‐2 Spike Protein via ECLIA

Electrochemiluminescence immunoassay (ECLIA) (Elecsys^®^ Anti-SARS-CoV-2 S, Roche Diagnostics, Rotkreuz, Switzerland) was performed according to the manufacturer’s instructions. Patient plasma is incubated with biotinylated and ruthenylated recombinant RBD or nucleocapsid antigens. After addition of streptavidin-coated microparticles, the resulting immune complexes are bound to the solid phase. The reaction mixture is transferred to the measuring cell and a chemiluminescence reaction is generated by applying a voltage. The emitted light is measured using Cobas E411 (Roche Diagnostics). Measured U/mL correlated strongly with the international WHO standard binding antibody units (BAU/mL) (U = 0.972 * BAU; Pearson r = 0.99996).

### 2.4 Jurkat cell line

The human T-cell line Jurkat clone E6-1 was stably transfected with the S-protein from the Wuhan variant of SARS-CoV-2 (Jurkat-S). An epHIV-7-based lentiviral vector was utilized containing the full-length S-protein sequences of the Wuhan variant, followed by an eGFP sequence, for eukaryotic expression (23, 24). To detect alloreactivity of plasma samples against Jurkat cells, non-transfected Jurkat cells (Jurkat-WT) were used as a control. Both, Jurkat-S and Jurkat-WT cells were cultured in complete Roswell Park Memorial Institute medium (RPMI), supplemented with 10% fetal calf serum (FCS), 100 U penicillin, and streptomycin (PAN-Biotech, Aidenbach, Germany). The cells were maintained in incubator at 37°C and 5% CO_2_.

### 2.5 Flow cytometric measurement of total IgG, IgG subclasses, IgM and IgA antibodies against SARS-CoV-2 spike protein using the spike protein-expressing Jurkat-S cells

Plasma samples from all time points were thawed on ice and centrifuged at 10,000× g in order to remove precipitates. Jurkat-WT cells were carboxyfluorescein succinimidyl ester (CFSE)-labeled by incubating cells with 5 µM carboxyfluorescein diacetate succinimidyl ester (CFDA-SE) for 20 min at room temperature in the dark. To analyze the relative binding of antibodies to Wuhan spike protein, a 1:2 mixture of 1 x 10^5^ Jurkat-S and CFSE-labeled Jurkat-WT cells were incubated for 20 min on ice in a 96 well V-bottom plate with either 10 µL of anticoagulated human plasma for the three-week time point or with 1:50 diluted plasma with phosphate-buffered saline (PBS) for the six-month time point. Thereafter, cells were centrifuged for 5 min at 400x g and washed two times with PBS. Subsequently, cells were resuspended in an antibody mixture containing 0.13 µg αIgG Fc : APC (clone M1310G05, BioLegend, San Diego, CA, USA), a 1:33 dilution of αIgA : PE-Vio 770 (clone REA1014, Miltenyi Biotec, Bergisch Gladbach, Germany), 0.5 µg αIgM : Spark Violet 423 (clone MHM-88), 0.5 µg αIgG2 Fc : PE (clone HP6002, SouthernBiotech, Birmingham, AL, USA), 0.75 µg αIgG3:PE (clone HP6050, BD Biosciences, Franklin Lakes, NJ, USA) and 1.25 µg αIgG4 Fc : AF647 (clone HP6025, SouthernBiotech) in a 1:4000 dilution of viability dye Zombie NIR (BioLegend) in PBS and incubated for 15 min at room temperature in the dark, followed by two additional washes with autoMACS Running Buffer (RB, Miltenyi Biotec). Lastly, we performed data acquisition using the Cytek^®^ Aurora flow cytometer operated with SpectroFlo software version 3.1.0 (Cytek Biosciences, Fremont, CA, USA). Subsequent analysis of flow cytometry data was performed using FlowJo software version 10.9 (FlowJo, Ashland, OR, USA).

Assuming that the ratio of the spike/eGFP proteins expressed in any given cell is a constant, we used the ratios of median fluorescence intensities (MFI) of anti-S antibodies to the MFI of eGFP as a direct estimate for the amounts of spike-specific plasma immunoglobulins of the various vaccinees. To mitigate batch effects resulting from different experimental runs, an early pandemic SARS-CoV-2 plasma sample was used for normalization. Blood donors demonstrating alloreactivity against the Jurkat-WT cells were identified via MFI ratios greater than twice the standard deviation of the mean for a given experimental run and were excluded from further analyses. To compensate for the different plasma dilutions at the two time points of venipuncture, we back-calculated the 1:50 dilution for all samples from the six-month time point to the undiluted relative concentrations using a 4PL sigmoidal fit. To this end, we used the plasma of one participant at the six-month time point, who had very high titers in all classes of Ig.

### 2.6 Neutralizing capacity against Wu‐Hu‐1 and B.1.1.529/BA.1 (Omicron)

The SARS‐CoV‐2 Surrogate Virus Neutralization Test (sVNT) kit (GenScript, Piscataway, NJ, USA) was used according to the manufacturer’s instructions. In short, frozen plasma was thawed, centrifuged at 10,000× g for 5 min to remove precipitates, and diluted 1:10 in dilution buffer. HRP peptides Wu‐Hu‐1 (SARS‐CoV‐2 spike protein (RBD, Avi and His Tag)‐HRP) and Omicron (SARS‐CoV‐2 spike protein RBD‐HRP, Omicron Variant, His Tag) (GenScript) were diluted at 1:1000. Diluted plasma samples and diluted HRP peptide samples were mixed in equal parts. After 30 min of incubation at 37°C, samples were pipetted onto ELISA capture plates and incubated for an additional 15 min at 37°C. Then, substrate solution was added and incubated for 15 min at room temperature before stop solution was added to terminate the reaction. Photometric measurements of the capture plate were performed at 450 nm using the InfiniteM200 (Tecan, Männeheim, Switzerland). Optical densities (OD) were used for calculation: Neutralizing Capacity = (1 − OD_sample_/OD_NegCtrl_) × 100%.

### 2.7 Anti‐human interferon gamma ELISpot

PBMCs were thawed and transferred to RPMI cell culture medium containing 1 mM pyruvate, 2 mM L‐glutamine, 10 mM HEPES, 10% FCS and 100 U penicillin/0.1 mg streptomycin (PAN‐Biotech). Cell count was performed by Cytek^®^ Aurora using SpectroFlo software version 3.1.0 (Cytek Biosciences). Live/dead differentiation was conducted in 3 μM 4′,6‐diamidino‐2‐phenylindole (DAPI; Biolegend). Three samples of 5 × 10^5^ PBMCs each were seeded into the wells of a polypropylene V‐bottom plate. The first sample remained untreated, the second and third were mixed with 0.2 μg of peptide pools representing either the Wu‐ Hu‐1 wild‐type strain (PepTivator^®^SARS‐CoV‐2 Prot_S B.1.1.529/BA.1 WT Reference Pool) or the Omicron variant of concern (PepTivator^®^ SARS‐CoV‐2 Prot_S B.1.1.529/BA.1 Mutation Pool) (Miltenyi Biotech). Subsequently, all samples were transferred to an anti‐ interferon gamma coated U‐bottom ELISpot plate (Human IFN‐gamma ELISpot Kit, R&D Systems, Minneapolis, MN, USA). Incubation was performed at 37°C and 5% CO_2_ for 24 hours. Thereafter, IFN-γ detection antibodies were added to each well, followed by incubation over night at 2 - 8°C. On the next day, alkaline phosphatase conjugated streptavidin was added and incubated for 2 h at room temperature in the dark. BCIP/NBT substrate was pipetted into each well, incubated for one more hour at room temperature, again in the dark. Plates were left to dry at 37°C for 30 min. Scanning and counting of the ELISpot plates was performed by ImmunoSpot 5.0 Analyzer with software version 5.0.9.15 (CTL Europe, Bonn, Germany).

### 2.8 *in vitro* re-stimulation and subsequent flow cytometric analyses

Frozen PBMCs were processed as described above. Two samples of 8 × 10^5^ PBMCs each were seeded in duplicates into 96‐well U‐bottom cell culture plates. One sample remained unstimulated throughout the experiment and served as a negative control. The second sample was stimulated with 1 μg of the vaccine BNT162b2 (Pfizer‐BioNTech, Mainz, Germany). After 20 h of incubation, 1 μg of brefeldin A (Sigma‐Aldrich) was added to all wells for another 4 h. After 24 h, cells were harvested, centrifuged at 400× g for 5 min, and duplicates were pooled in autoMACS Running Buffer (Miltenyi Biotec). Subsequently, cells were washed with PBS, centrifuged for 5 min at 400× g and incubated with 1:2000 diluted Zombie NIR (BioLegend) for 20 min at room temperature in darkness. Thereafter, non‐specific binding sites were blocked with 2.5 μL of FCS (Fisher Scientific, Pittsburgh, PA, USA), 1.25 μL of True‐Stain Monocyte™ Blocker, and 1.25 μL of Human TruStain FcX™ (BioLegend) and incubated for 15 min on ice in darkness. The following antibody:fluorophore combinations were used for surface staining per sample: 0.125 µg CD3:FITC (clone UCHT1), 0.0156 µg CD4:BV750 (clone SK3), 0.0625 µg CD8:BV570 (clone RPA-T8), 0.125 µg CD25:APC (clone BC96), 0.25 µg CD27:BV605 (clone O323), 0.125 µg CD45RA : APC-Fire750 (clone HI100), 0.125 µg CD45RO : BV421 (clone UCHL1), 1 µg Fas-L:PE (clone NOK1) (BioLegend) and 2 µL CD137:PE-Vio615 (clone REA765) (Miltenyi Biotec). After incubation on ice for 15 min in darkness, samples were fixed with fixation buffer (BioLegend) for 20 min at room temperature in darkness, followed by three washes with intracellular staining and permeabilization wash buffer (BioLegend). Subsequently, non‐specific binding sites were again blocked as described above. For intracellular cytokine staining, the following antibody:fluorophore combinations were used per sample: 0.5 µg IFN-γ:PerCP-Cy5.5 (clone 4S.B3), 0.125 µg IL-2:BV650 (clone MQ1-17H12), and 1 µg TNFα:PE-Cy7 (clone Mab11) (BioLegend). After 30 min of incubation at room temperature in darkness, a final wash with intracellular staining and permeabilization wash buffer was performed before samples were measured by flow cytometry using Cytek^®^ Aurora with SpectroFlo software version 3.1.0 (Cytek Biosciences).

### 2.9 Statistics

Contingency table analyses were performed via chi-square test. Data were tested for Gaussian distribution using the Shapiro-Wilk test. Comparison of independent data following Gaussian distribution were performed via one-way analysis of variance (ANOVA) for three groups with Tukey-Kramer correction for multiple comparisons. Pair‐wise comparisons of data not following Gaussian distribution were performed via Wilcoxon matched‐pairs signed rank test for two groups. Unpaired comparisons of three groups were performed via Kruskal–Wallis followed by Dunn´s correction for multiple comparisons. Correlation analyses were performed via Spearman rank correlation. Statistical assays were performed with GraphPad InStat^®^ version 3.10 for Windows (GraphPad Software, San Diego, CA, USA) or IBM SPSS Statistics Version 27 (IBM, Armonk, NY, USA). Graphs were created with SigmaPlot 13.0 (Inpixon, Palo Alto, CA, USA).

### 2.10 Ethic commitment

This study was approved by the ethics committee of the Rostock University Medical Center under the file number A 2020‐0086. Written informed consent was provided by all participants.

## 3 Results

### 3.1 Study design

For our study, we recruited 79 subjects from both, the local university medical center and the municipal vaccination center. 32 of these subjects donated blood via venipuncture at day 0, before the first vaccination, to serve as a control. Blood samples were taken from all study participants on days 21 and approximately six months following the first immunization. In detail, 42 study participants who received NVX-CoV2373 (Novavax), 19 who received AZD1222 (AstraZeneca) and 18 who received BNT162b2 (BioNTech) as their first vaccine were available for venipuncture on day 21. At approximately day 180, 28 participants were available who had obtained two immunizations with NVX-CoV2373. Only one participant had obtained two immunizations with AZD1222 and was available at 6 months. The remaining 18 who had obtained AZD1222 as their first vaccine obtained BNT162b2 as their second. They were available at 6 months as were the 18 who had received BNT162b2 as their first and second vaccine. At the time of the first venipuncture, seven study participants in the Novavax group tested positive for anti-nucleocapsid antibodies and were thus defined as already recovered. By the time of the second venipuncture, nine additional subjects in the Novavax group were tested positive for anti-nucleocapsid antibodies and therefore also considered recovered. All vaccination groups were comparable with respect to gender (p = 0.807; Chi² test) and age (p = 0.112, Kruskal-Wallis test) of the participants. We have provided an overview of all study participants, their demographic data in in **Supplementary Table 1** and their COVID-19 history in **Supplementary Table 2**.

### 3.2 Early humoral immune response was most pronounced following vaccination with BNT162b2

**Figure 1:**
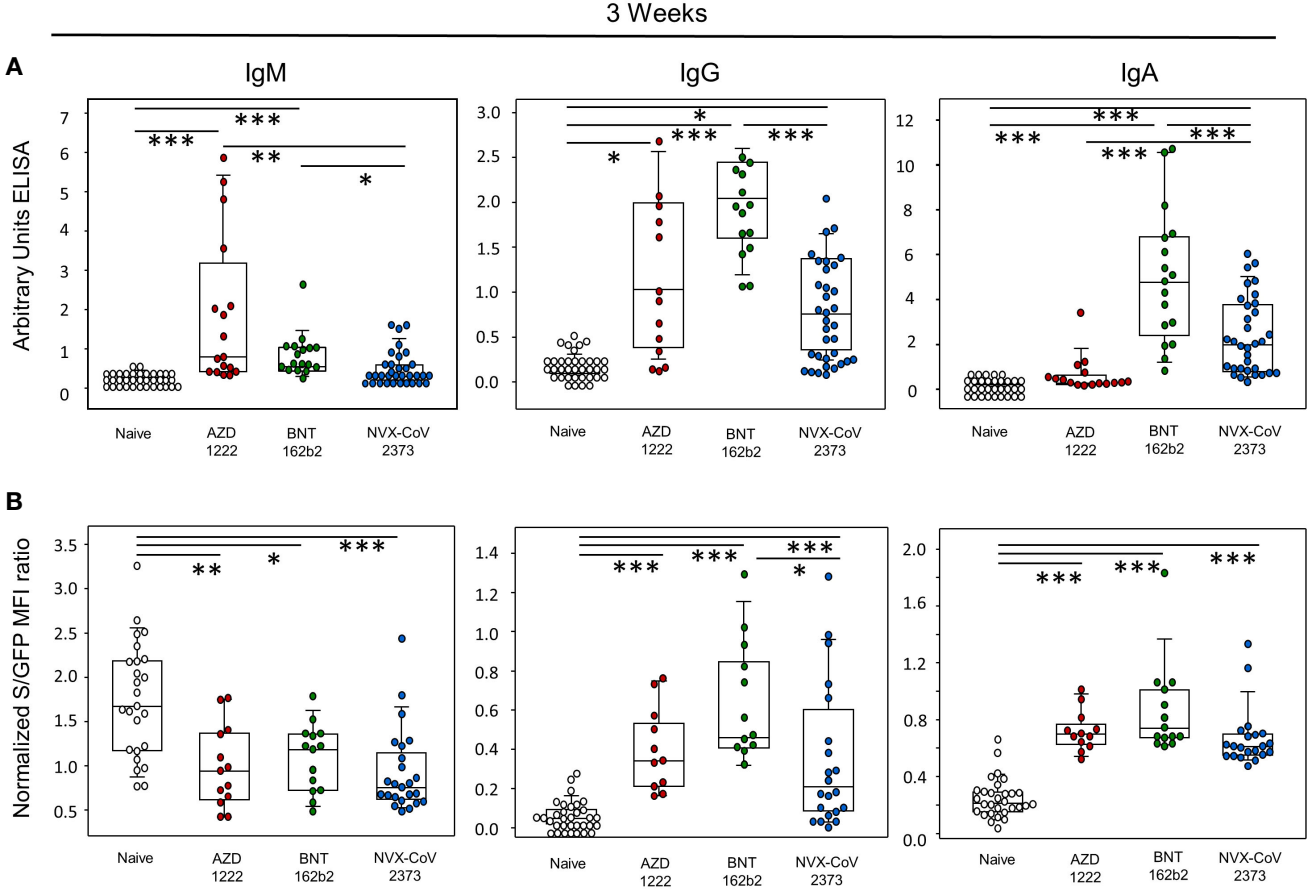
Early humoral immune response was most pronounced following vaccination with BNT162b2. **(A)** Determination of IgM, IgG and IgA antibodies by enzyme-linked immunosorbent assay (ELISA) as arbitrary units. **(B)** determination of IgM, IgG, and IgA antibodies as MFI ratio using SARS-CoV-2 spike protein-expressing Jurkat-S cells; data represent day 21 without vaccination (naïve control; white dots) or after a single vaccination with AZD1222 (AstraZeneca; red dots), BNT162b2 (Pfizer-BioNTech; green dots), NVX-CoV2373 (Novavax; blue dots). Boxplots show medians with 25 and 75 percentiles, each dot represents one study participant. *p-values resulted from Kruskal-Wallis tests followed by Dunn´s correction for multiple comparisons: *p<0.05; **p<0.01; ***p<0.001.

In order to investigate the early humoral immune responses towards the three vaccines 21 days after initial vaccination, we excluded all individuals who at this time had already had contact with SARS-CoV-2 and assessed IgM, IgG and IgA titers via ELISA. As shown in **Figure 1A**, a single dose of AZD1222 and BNT 162b2 yielded significantly more IgM than NVX-CoV2373, BNT162b2 yielded significantly more IgG than NVX-CoV2373, and NVX-CoV2373 and BNT162b2 were superior to AZD1222 with respect to IgA. Compared to the vaccine-naive control group, only BNT162b2 produced significantly more antibodies for all immunoglobulins. AZD1222 only significantly outperforms the control group with regard to IgM and IgG, NVX-CoV2373 only with regard to IgA and IgG. Since recombinant spike protein fragments cannot reproduce the native quaternary structure of the protein trimer, we therefore used a spike protein expressing human T cell line, Jurkat clone E6-1, to measure antibody binding against the wild type Wu-Hu-1. This assay resulted in significantly more binding of the spike protein by IgG antibodies from study participants who had obtained BNT162b2 compared to those with NVX-CoV2373 (**Figure 1B**). Compared to the control group, the spike protein binds significantly more immunoglobulins from all vaccination groups, with the exception of IgM.

### 3.3 At six months after primary immunization, most antibody levels resulting from NVX-CoV2373 vaccination were exceeded by vaccination involving BNT162b2 and by infection

**Figure 2:**
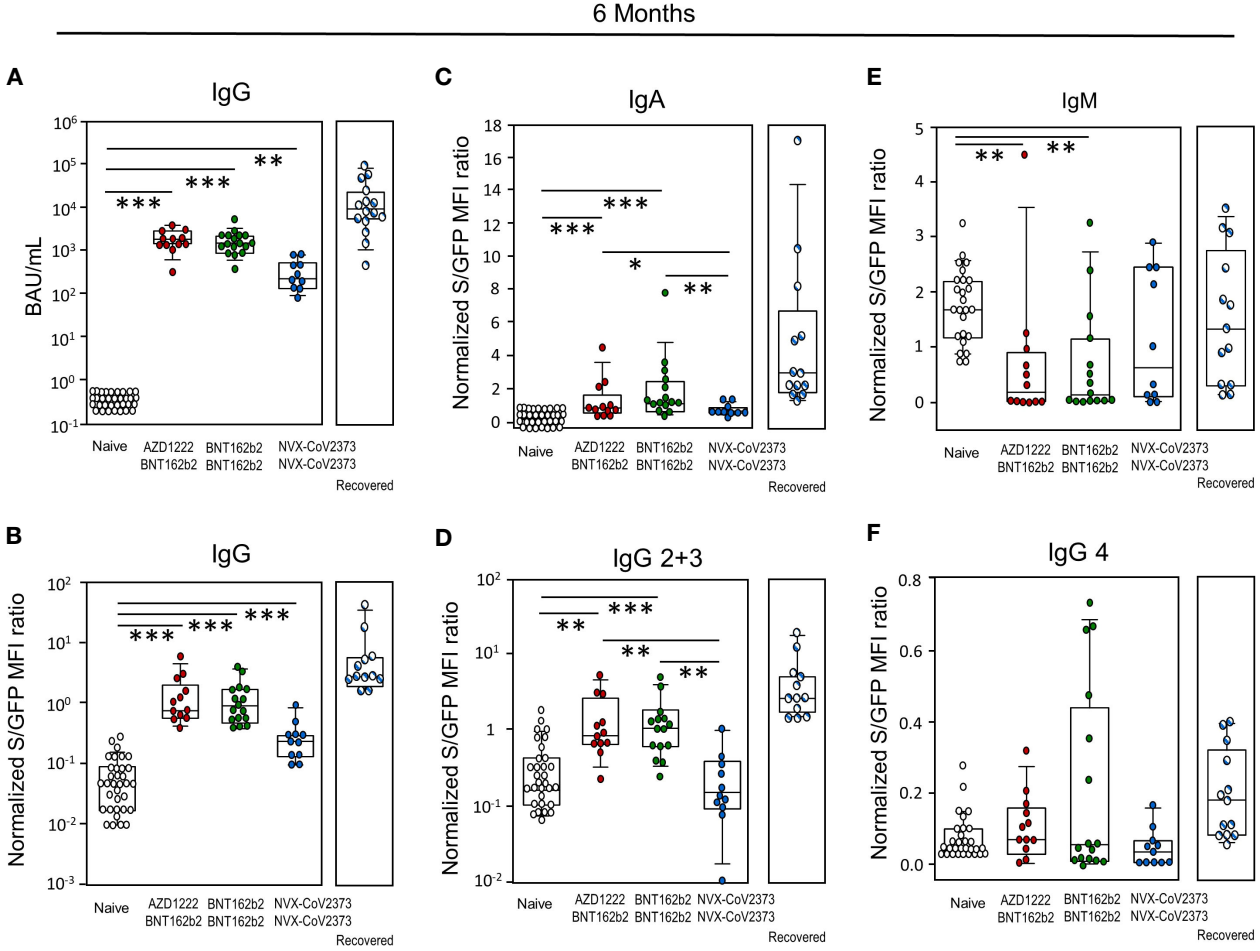
Antibody levels resulting from NVX-CoV2373 vaccination were exceeded by both, infection and immunization involving BNT162b2. **(A)** Determination of total IgG antibodies by electrochemiluminescence immunoassay (ECLIA) as BAU/mL and **(B)** as MFI ratio using SARS-CoV-2 spike protein expressing Jurkat-S cells. **(C)** Determination of IgA, **(D)** IgG2 + 3, **(E)** IgM and **(F)** IgG4 antibodies as MFI ratio using SARS-CoV-2 spike protein expressing Jurkat-S cells; data represent approximately day 180 after the primary immunization. The type of secondary vaccine is indicated – either NVX-CoV2373 or BNT162b2. Boxplots show medians with 25 and 75 percentiles, each dot represents one study participant, striped dots indicate study participants immunized with NVX-CoV2373 and recovered from COVID-19. *p-values resulted from Kruskal-Wallis tests and Dunn´s correction for multiple comparisons: *p<0.05; **p<0.01; ***p<0.001.

At six months, the total amount of spike-specific RBD-IgG was quantified via ECLIA and Jurkat-S cells, respectively. The positive trend observed for BNT162b2 at three weeks extended to this time point as both, homologous and heterologous vaccination with BNT162b2 resulted in significantly more BAU/mL with medians of 1781 (AZD1222/BNT162b2), 1537 (BNT162b2/BNT162b2) and 251 (NVX-CoV2373/NVX-CoV2373) for participants, who had not yet recovered from SARS-CoV-2. For those NVX-CoV2373 vaccinees who had already recovered from infection, the median was 9212 BAU/ml (**Figure 2A**). Likewise, MFI for total IgG were higher after vaccinations involving BNT162b2 (**Figure 2B**) and again, previous contact with the virus led to a significantly higher MFI of 2.78. All vaccination groups remain at significant higher level of IgG compared to the control group independent from the technical method. When assessing differences in IgA and IgG isotypes elicited by the various vaccines, BNT162b2 - either alone or in combination with AZD1222 - yielded significantly more IgA, IgG2 and IgG3 antibodies compared to the homologous regime with NVX-CoV2373 and the control group (**Figure 2C, D**). Significant differences were found for IgM in the control group compared to homologous and heterologous vaccination with BNT162b2, none for IgG4 (**Figure 2E, F**). Additional contact with SARS-CoV-2 due to infection led to significantly increased binding of the Jurkat-S cells and that was true for total IgG, all tested IgG subclasses, as well as IgA. Transient expression of IgM rendered its expression difficult to evaluate (**Figure 2B-F**).

### 3.4 Neutralization capacity was comparable among the three vaccination groups yet was exceeded by additional infection

**Figure 3:**
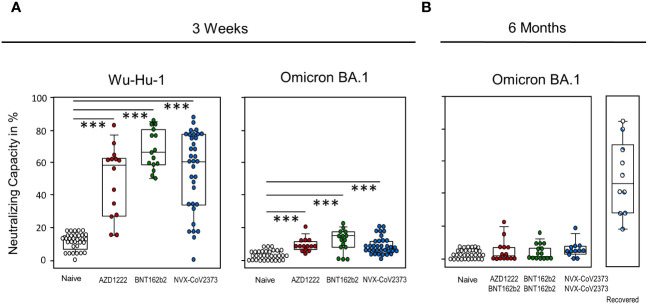
Neutralization capacity is comparable among the three vaccination groups yet is exceeded by additional infection. Neutralizing capacity was assessed via surrogate virus neutralization test (sVNT) against **(A)** wild-type Wu-Hu-1 and Omicron BA.1 on day 21 after single vaccination with NVX-CoV2373, AZD1222, BNT162b2 or without vaccination. **(B)** Neutralizing capacity against Omicron BA.1 was assessed at six months after the first vaccination and after having received a second dose - either NVX-CoV2373 or BNT162b2. Boxplots show medians with 25 and 75 percentiles, each dot represents one study participant. Striped dots indicate study participants immunized with NVX-CoV2373 and recovered from COVID-19. *p-values resulted from one way analysis of variance (ANOVA) followed by Tukey-Kramer correction for multiple comparisons: ***p<0.001.

To test for humoral functionality, we performed surrogate virus neutralization tests. At day 21, Neutralization against wild-type Wuhan (Wu-Hu-1) differs from that of Omicron BA.1, but is comparable between the groups, with BNT162b2 vaccinees again tending to achieve a slight advantage over the two other vaccines. All vaccination groups significantly surpass the control group for both spike protein variants. At six months, there is no discernible difference between vaccinated and unvaccinated people anymore, whereas additional contact with SARS-CoV-2 due to infection augmented the neutralizing capacity significantly (**Figure 3B**). **Supplementary Table 3** shows that neutralizing capacity correlated strongly with ELISA results.

### 3.5 NVX-CoV2373 induced short-term and long-term T cell memory

**Figure 4:**
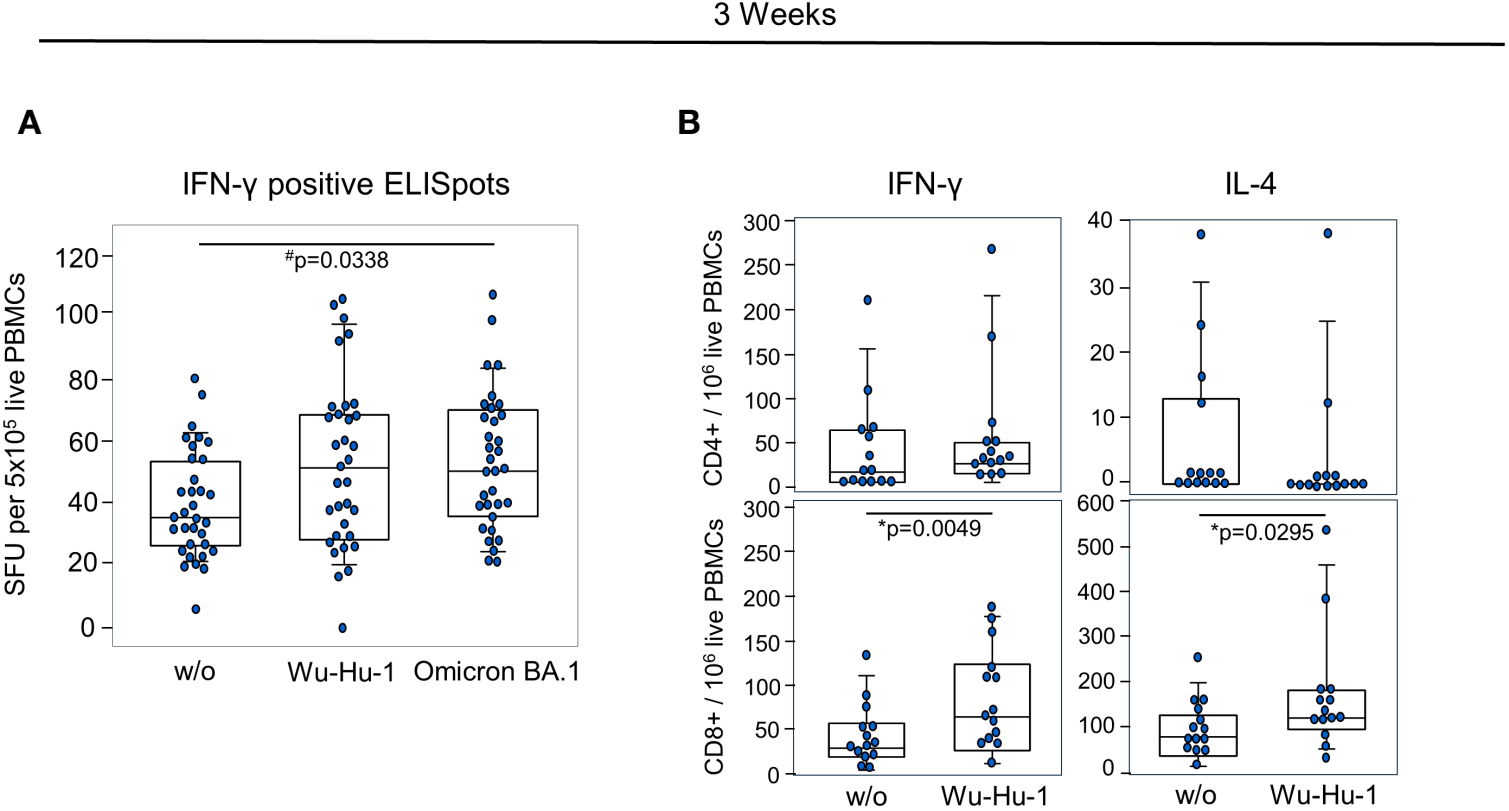
NVX-CoV2373 induces short-term T cell memory. **(A)** Combinations of dot- and box plots show absolute numbers of IFN-γ positive Spot forming units (SFU) resulting from ELISpot using 5x10^5^ PBMCs either without any stimulation (w/o) or stimulated with peptide pools representing the Wu-Hu-1 or the Omicron BA.1 spike protein. **(B)** Combinations of dot- and box plots show the numbers of IFN-γ and IL-4 producing CD4+ T helper (upper panel) and CD8+ cytotoxic T cells (lower panel) after *in vitro* re-stimulation with the BNT162b2 vaccine. #p-values resulted from one-way ANOVA followed by Tukey-Kramer correction for multiple comparisons and *Wilcoxon matched-pairs signed-ranks test. Assays were performed on day 21 after primary vaccination.

To assess the early T-cell response against the NVX-CoV2373 vaccine, we performed an ELISpot at day 21. *In vitro* re-stimulation with two peptide pools mapping either the wild-type (Wu-Hu-1) or the Omicron BA.1 spike protein yielded significant increases in IFN-γ production. While there was a median of 35 spots in the absence of peptide stimulation, there were medians of 47 and 50 after stimulation with Wu-Hu-1 and Omicron BA.1 variant mapping peptides, respectively (**Figure 4A**). Flow cytometric analyses confirmed the IFN-γ production for CD8+ cytotoxic T cells and revealed a significant increase in IL-4 production of CD8+ cells (**Figure 4B**).

**Figure 5:**
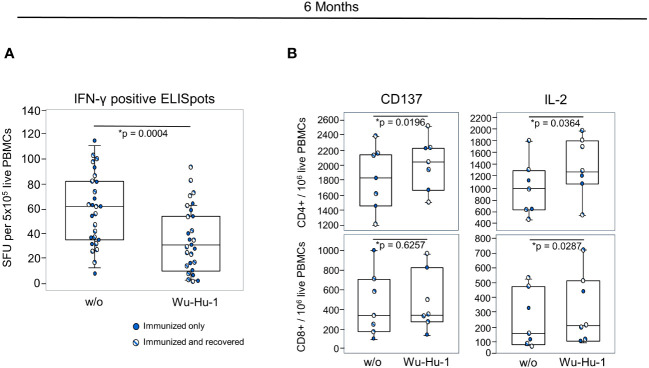
NVX-CoV2373 induces long-term T cell memory. **(A)** Combinations of dot- and box plots show absolute numbers of IFN-γ positive SFU resulting from ELISpot using 5x10^5^ PBMCs either without any stimulation (w/o) or stimulated with a peptide pool representing the Wu-Hu-1 spike protein. **(B)** Combinations of dot- and box plots show the numbers of CD137+ and IL-2 producing CD4+ T helper (upper panel) and CD8+ cytotoxic T cells (lower panel) after *in vitro* re-stimulation with the BNT162b2 vaccine. Gating was on CD45RO+ CD45RA- CD27+ memory cells as exemplified in the **Supplementary Figure 1**. Blue dots represent vaccinated participants, striped dots indicate study participants immunized with NVX-CoV2373 and recovered from COVID-19. *p-values resulted from paired t-tests. In Figure B only, participants who had recovered from COVID-19 were included for the statistical calculation. Assays were performed approximately six months after primary vaccination.

To assess the later T cell response against the NVX-CoV2373 vaccine and to investigate the impact of previous contact with the virus, we performed another ELISpot at six months. At that time point, we no longer detected an increased IFN-γ production (**Figure 5A**). However, we observed a significant increase in the activation marker CD137 on CD4+/CD45RO+/CD45RA-/CD27+ memory cells measured via flow cytometry. In addition, *in vitro* re-stimulation with BNT162b2 led to a significant increase in IL-2 production in CD4+ and CD8+ T cells, alike (**Figure 5B**). In contrast to the humoral response, additional contact with SARS-CoV-2 due to infection had no significant impact on the T cell memory. An overview of all measured markers and cytokines for both timepoints is provided in the **Supplementary Tables 4 and 5**.

## 4 Discussion

We here demonstrated a cellular immune memory induced by NVX-CoV2373 at both, three weeks and six months after primary immunization. At three weeks, there was a 1.3- and 1.4-fold increase in IFN-γ positive ELISpots following Wu-Hu-1 and Omicron BA.1 peptide specific re-stimulation, respectively. And even though we previously quantified and published the T cell response following AZD1222 and BNT162b2 vaccination, a direct comparison between the three vaccination groups with respect to the T cell memory is difficult. While in our earlier study we compared day 0 to day 21, we here looked at day 21 only and compared ELISpots in the absence of stimulation to those following peptide exposure. However, the range of increase in IFN-γ positive ELISpots is comparable with 1.3 for NVX-CoV2373, 1.4 for AZD1222 and 2.1 for BNT162b2 (25). Why at six months we found good IFN-γ responses resulting from ELISpot following BNT162 and AZD1222 immunizations (9) yet hardly any following NVX-CoV2373, we cannot explain. We can only speculate that the various vaccines induced different types of memory T cells as shown previously (26). Of note though, infection with SARS-CoV-2 did not add to the NVX-CoV2373 induced T cell memory, neither for the ELISpot, nor for recall cytokine production measured via flow cytometry. As for the latter, we used the BNT162b2 vaccine for re-stimulation in order to improve antigen presentation via the HLA class I molecules. And indeed, while others, who used peptide pools, presented predominantly CD4+ responders (26–28), we here obtained significant IFN-γ and IL-4 responders among CD8+ cytotoxic T cells at three weeks. At six months, this observation was reversed and we also observed a larger fraction of memory T cells and IL-2 producers among the CD4 T helper than CD8 cytotoxic T cells. Interestingly, we observed significant IL-2 memories among both, CD4 and CD8 T cells at 12 months following AZD1222 and BNT162b2 vaccination with even higher numbers for the CD8 cells (13). This may suggest that vector and mRNA vaccines are better suited to induce CD8 memories and could thus facilitate a more effective immune response to COVID-19 infections (29–31). However, a direct comparison with samples taken and processed at similar dates is still pending. Since our ELISpot and the cellular response measured by flow cytometry are well differentiated, but relatively low, and also innate stimuli cannot be excluded, our results must be interpreted with caution.

As for the humoral immune response to NVX-CoV2373 and its comparison to BNT162b2 and AZD1222, the mRNA vaccine induced the strongest antibody responses at both time points, three weeks and six months. This finding was true for total IgG, all tested IgG isotypes, and IgA and was independent of the assay used – an ELISA detecting antibodies binding to an S1 domain of the spike protein on a two-dimensional microtiter plate, an ECLIA detecting antibodies binding the receptor binding domain, or the Jurkat-S cell that allows binding to the native quaternary structure of the spike protein trimer. Our data thus confirm and extend on a previous publication using an ELISA only, to show that NVX-CoV2373 is slightly inferior in terms of antibody levels evoked (26). However, we cannot explain why vaccination naïve sera showed the highest Jurkat-S binding via IgM antibodies. We would like to suggest technical reasons but the fact that later time points and other isotypes mirror the results from both, ELISA and ECLIA refute this option. Even though antibody levels have frequently been shown to correlate with neutralization capacities (32–34), we here show that the antibodies resulting from the BNT162b2 vaccine only trended towards improved neutralization, at least for the Wu-Hu-1 and Omicron BA.1 strain. For the sVNT we used, a strong correlation - at least for the wild type - with true Virus Neutralization Test has been described (35), suggesting a small advantage for BNT162b2 vaccinees in terms of potential protection, unless the validity for current mutation variants is refuted. As for the variants, not only we but also others observed comparable neutralization capacities for all three vaccines (26, 27, 36, 37). Real world data on the efficacy of the various vaccines would require a look at infection rates among the three vaccination groups – beyond the two to three months analyzed in phase 3 clinical studies (3, 5, 21, 22). However, this comparison is inadequate as NVX-CoV2373 was not used in Germany until spring of 2022, when many had already recovered from at least one infection. Moreover, at that time the original Wu-Hu-1 strain had been replaced by variants. Indeed, our AZD1222 and BNT162b2 cohorts were collected in the spring and summer 2021 when the dominant strain was Alpha or Delta, so no infections have occurred up to this point. In contrast, the NVX-CoV2373 cohort was recruited during the Omicron wave in the spring of 2022. Of note, while additional infections did not seem to add to the T cell memory, the humoral immune response was clearly boosted by encounter with the virus.

There are limitations to our study. i) our cohorts are rather small and we only recorded sex, age and infection status. No information on health status or chronic diseases is known, which might influence any results. ii) because the cohorts were recruited more than a year apart, plasma of the former cohorts had been frozen correspondingly long. Moreover, only limited amounts of patient material had been preserved, so that some plasma and –most importantly PBMC - had not been available for direct comparisons. Because we are limited by the small sample size for cellular investigation at six months, the results should be understood more as indicative. Single cell analysis needs to be performed on a larger scale to obtain a long-term immunological profile of NVX-CoV2373 vaccinated individuals.

In summary, our results suggest that all vaccines investigated here are comparably efficacious, with slight advantages i) for the humoral response towards the mRNA vaccine and possibly ii) for a CD8 memory towards vector and mRNA-based vaccines. However, whether these slight differences translate into real world protection from infection, mitigation of severe disease courses and prevention of long/post COVID will need to be investigated in detail. Moreover, the low numbers of NVX-CoV2373 vaccines administered – less than 2% of the approximately 192 million vaccines administered in Germany are NVX-CoV2373 – precludes an adequate comparison in terms of frequency and severity of adverse events (https://de.statista.com/infografik/30627/in-deutschland-verabreichte-coroana-impfungen/). The recent approval of monovalent vaccines from BioNTech, Moderna, and Novavax adapted to the currently dominant variant Omicron XBB.1.5 will allow for in-depth follow-ups.

## Data availability statement

The original contributions presented in the study are included in the article/**Supplementary Material**. Further inquiries can be directed to the corresponding author.

## Ethics statement

The studies involving humans were approved by Ethics committee of the Rostock University Medical Center. The studies were conducted in accordance with the local legislation and institutional requirements. Written informed consent for participation in this study was provided by the participants’ legal guardians/next of kin.

## Author contributions

FM: Data curation, Formal Analysis, Investigation, Methodology, Software, Validation, Visualization, Writing – original draft, Writing – review & editing. MK: Methodology, Writing – review & editing. WB-E: Methodology, Writing – review & editing. ER: Conceptualization, Funding acquisition, Project administration, Resources, Supervision, Writing – review & editing. BM-H: Conceptualization, Data curation, Formal Analysis, Project administration, Resources, Software, Supervision, Validation, Visualization, Writing – original draft, Writing – review & editing.

## Supplementary material

Supplementary Figure 1 Pseudo-color plots exemplify the strategy employed for gating on CD4+ and CD8+ memory T cells for subsequent intracellular cytokine analysis. After inclusion of single cells and exclusion of dead cells, CD3+ T cells were gated upon and differentiated according to CD8+ expression as cytotoxic and CD4+ expression as T helper cells. CD45RO and CD27 expression then defined memory cells that were subsequently analyzed for CD137 and IL-2 expression.
