# Supplementary material for: NVX-CoV2373 induces humoral and cellular immune responses that are functionally comparable to vector and mRNA-based vaccines
Source: Front Immunol. 2024 Mar 18;15:1359475. doi: 10.3389/fimmu.2024.1359475 (PMC10982398; doi:10.3389/fimmu.2024.1359475)
Supplement: Supplementary file 1 [file DataSheet_1.docx]

Supplementary Material

## Supplementary Figures

Supplied in section of regular figures, as requested.

**
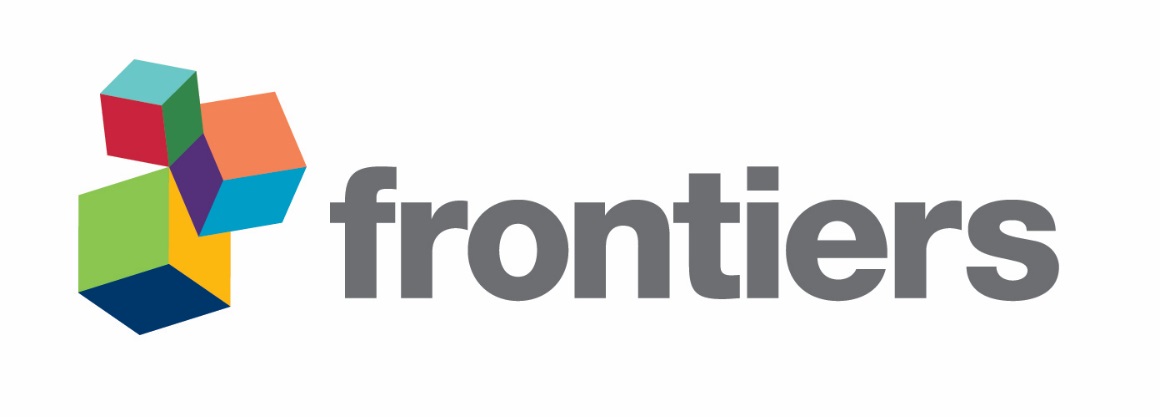
**

**Supplementary Figure 1.** Pseudo-color plots exemplify the strategy employed for gating on CD4+ and CD8+ memory T cells for subsequent intracellular cytokine analysis. After inclusion of single cells and exclusion of dead cells, CD3+ T cells were gated and differentiated according to CD8+ expression as cytotoxic and CD4+ expression as T helper cells. CD45RO and CD27 expression then defined memory cells that were subsequently analyzed for CD137 and IL-2 expression.

## Supplementary Tables

**Supplementary Table 1.** Demographic data of study participants.

|  | NVX-CoV2373  (n = 42) | AZD1222  (n = 19) | BNT162b2  (n = 18) | Vaccination naive  (n = 32) | p-value |
| --- | --- | --- | --- | --- | --- |
| Male/Female [n] | 20/22 | 9/10 | 6/11 | 13/19 | 0.807^*^ |
| Median Age [Years]  (Min/Max) | 44  (21/68) | 38  (21/58) | 38  (24/60) | 38  (21/58) | 0.112^#^ |

* Chi²-Test; # Kruskal-Wallis-Test;

**Supplementary Table 2.** COVID-19 history of all study participants.

| Subject | Self-reported disease | Nucleocapsid Antibodies  > 1 = positive |  | Subject | Self-reported disease | Nucleocapsid Antibodies  > 1 = positive |
| --- | --- | --- | --- | --- | --- | --- |
| N1  N2  N3  N4  N5  N6  N7  N8  N9  N10  N11  N12  N13  N14  N15  N16  N17  N18  N19  N20  N21  N22  N23  N24  N25 | No  Yes  No  No  No  No  No  No  No  No  No  No  No  No  No  No  No  No  No  No  No  No  No  No  No | 0.08  0.26  0.07  0.08  0.08  0.13  0.08  0.07  0.08  10.92  0.08  0.08  0.08  0.07  0.08  0.07  0.08  0.09  106.2  0.07  0.07  0.08  0.08  0.07  14.06 |  | A2  A3  A4  A5  A6  A7  A8  A9  A10  A11  A12  A13  A14  A15  A16  A17  A18  A19  A20  B0  B1  B2  B3  B4  B5 | No  No  No  No  No  No  No  No  No  No  No  No  No  No  No  No  No  No  No  No  No  No  No  No  No | 0.09  0.11  0.09  0.09  0.09  0.09  0.09  0.09  0.10  0.09  0.09  0.09  0.06  0.10  0.09  0.12  0.07  0.09  0.09  0.07  0.07  0.09  0.09  0.09  0.15 |
| Subject | Self-reported disease | Nucleocapsid Antibodies  > 1 = positive |  | Subject | Self-reported disease | Nucleocapsid Antibodies  > 1 = positive |
| N26  N27  N28  N29  N30  N31  N32  N33  N34  N35  N36  N37  N38  N39  N40  N41  N42  C1  C2  C3  C4  C5  C6  C7  C8 | No  No  No  No  No  No  No  Yes  Yes  No  No  No  No  No  No  No  No  No  No  No  No  No  No  No  No | 0.08  0.07  0.09  0.08  0.08  24.54  0.08  20.80  6.52  0.07  0.08  0.08  0.07  0.08  0.08  0.08  0.08  0.09  0.11  0.09  0.09  0.09  0.09  0.09  0.09 |  | B7  B8  B9  B10  B11  B13  B14  B15  B17  B18  B19  B20  C15  C16  C17  C18  C19  C20  C21  C22  C23  C24  C25  C26  C27 | No  No  No  No  No  No  No  No  No  No  No  No  No  No  No  No  No  No  No  No  No  No  No  No  No | 0.10  0.10  0.09  0.11  0.08  0.09  0.10  0.09  0.09  0.14  0.10  0.10  0.12  0.09  0.09  0.09  0.09  0.09  0.15  0.10  0.10  0.09  0.11  0.08  0.09 |
| Subject | Self-reported disease | Nucleocapsid Antibodies  > 1 = positive |  | Subject | Self-reported disease | Nucleocapsid Antibodies  > 1 = positive |
| C9  C10  C11  C12  C13  C14 | No  No  No  No  No  No | 0.10  0.09  0.09  0.09  0.10  0.10 |  | C28  C29  C30  C31  C32  C33 | No  No  No  No  No  No | 0.10  0.09  0.09  0.10  0.09  0.09 |

N = NVX-CoV2373 (3 Weeks); A = AZD1222 (3 Weeks); B = BNT162b2 (3 Weeks); C = Vaccination naïve (@ first Vaccination)

**Supplementary Table 3.** Antibody units of all isotypes correlate with neutralizing capacity against Wuhan-Hu-1 on day 21 after a single dose of vaccine.

|  | NVX-CoV2373 | | AZD1222 | | BNT162b2 | |
| --- | --- | --- | --- | --- | --- | --- |
|  | r* | p-value* | r* | p-value* | r* | p-value* |
| IgM | 0.546 | 0.009 | 0.823 | 0.0003 | 0.646 | 0.009 |
| IgG | 0.809 | 0.00005 | 0.841 | 0.0003 | 0.952 | < 0.00001 |
| IgA | 0.708 | 0.0002 | 0.820 | 0.0003 | 0.536 | 0.04 |

* = Spearman rank correlation

**Supplementary Table 4.** In vitro re-stimulation of PBMC with BNT162b2 yielded an increase in IFN-γ and IL-4 producing cytotoxic T cells on day 21 after a single dose of NVX-CoV2373.

|  | non-stimulated | stimulated | p-value* |
| --- | --- | --- | --- |
| **CD4+ T-helper cells** | positive cells/10^6^ PBMC [median of n] | |  |
| IFN-γ | 11 | 21 | 0.311 |
| GrzB | 3730 | 3562 | 0.245 |
| IL-2 | 37 | 38 | 0.271 |
| IL-4 | 0 | 0 | 0.180 |
| IL-10 | 656 | 579 | 0.221 |
| TNFα | 377 | 326 | 0.331 |
| CD137 | 596 | 714 | 0.826 |
| Fas-L | 1295 | 991 | 0.096 |
| **CD8+ cytotoxic T cells** |  |  |  |
| IFN-γ | 24 | 60 | 0.008 |
| GrzB | 60400 | 59007 | 0.030 |
| IL-2 | 140 | 180 | 0.221 |
| IL-4 | 87 | 130 | 0.030 |
| IL-10 | 619 | 545 | 0.594 |
| TNFα | 214 | 168 | 0.363 |
| CD137 | 161 | 151 | 0.510 |
| Fas-L | 266 | 228 | 0.600 |

* Wilcoxon matched pairs signed rank test

**Supplementary Table 5.** In vitro re-stimulation of PBMC with BNT162b2 yielded an increase in CD137 and IL-2 producing memory cells at six months after primary immunization.

|  | non-stimulated | stimulated | p-value* |
| --- | --- | --- | --- |
| **CD4+ T-helper cells** | positive cells/10^6^ PBMC [median of n] | |  |
| IFN-γ | 4918 | 4566 | 0.176 |
| IL-2 | 525 | 674 | 0.091 |
| TNFα | 450 | 451 | 0.398 |
| CD137 | 3856 | 3889 | 0.310 |
| Fas-L | 3177 | 2951 | 0.051 |
| CD45RO+ CD45RA- CD27+ IL-2+ | 997 | 1280 | 0.018 |
| CD45RO+ CD45RA- CD27+ CD137+ | 1837 | 2048 | 0.028 |
| **CD8+ cytotoxic T-cells** |  |  |  |
| IFN-γ | 1900 | 2067 | 0.499 |
| IL-2 | 129 | 142 | 0.735 |
| TNFα | 519 | 584 | 0.499 |
| CD137 | 1601 | 1583 | 0.735 |
| Fas-L | 1154 | 922 | 0.176 |
| CD45RO+ CD45RA- CD27+ IL-2+ | 152 | 206 | 0.028 |
| CD45RO+ CD45RA- CD27+ CD137+ | 344 | 345 | 0.499 |

* Wilcoxon matched pairs signed rank test
