# Supplementary figures and images for: NVX-CoV2373 induces humoral and cellular immune responses that are functionally comparable to vector and mRNA-based vaccines
Source: Front Immunol. 2024 Mar 18;15:1359475. doi: 10.3389/fimmu.2024.1359475 (PMC10982398; doi:10.3389/fimmu.2024.1359475)

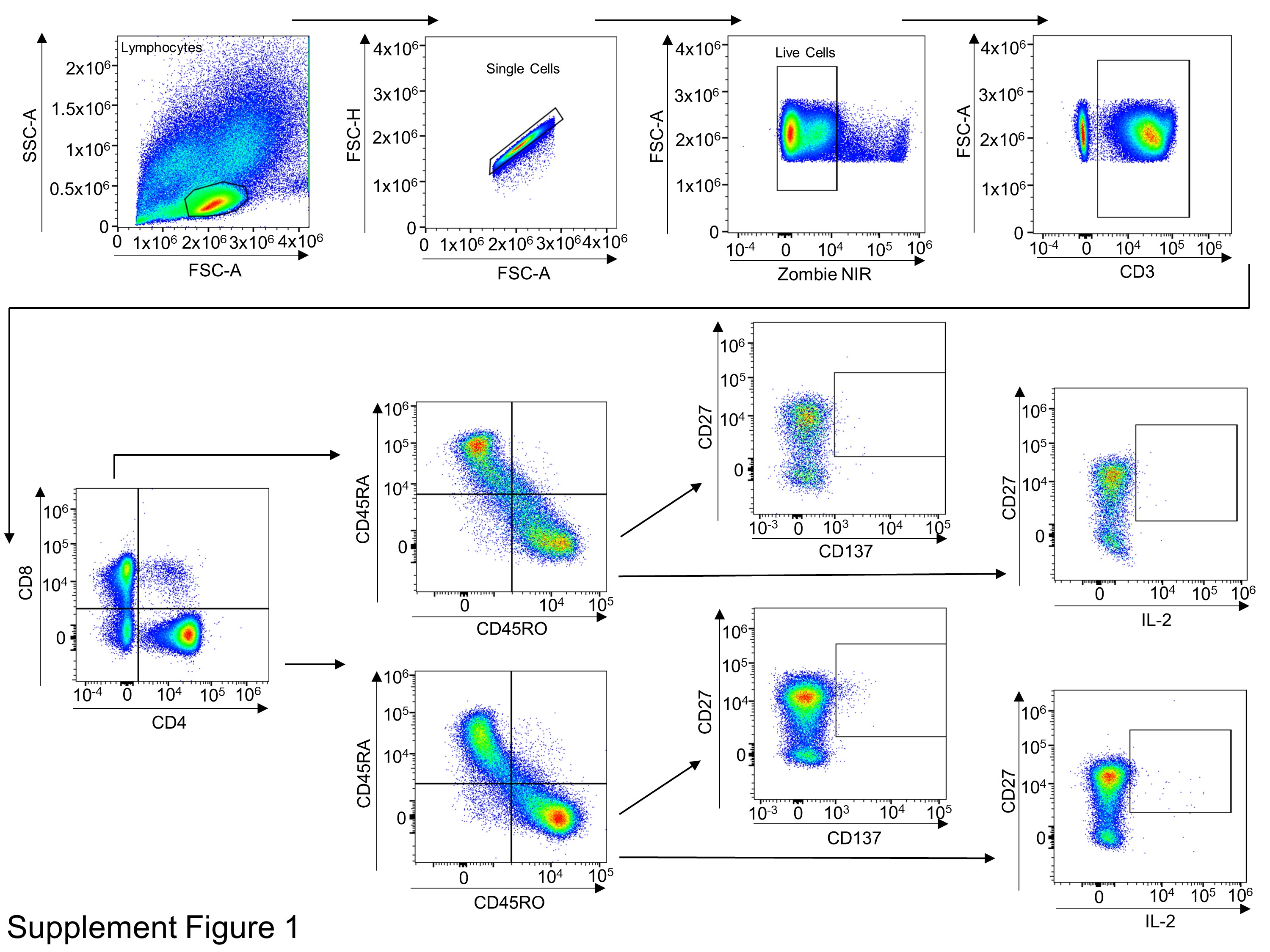

Supplement: Supplementary Figure 1 — Pseudo-color plots exemplify the strategy employed for gating on CD4+ and CD8+ memory T cells for subsequent intracellular cytokine analysis. After inclusion of single cells and exclusion of dead cells, CD3+ T cells were gated upon and differentiated according to CD8+ expression as cytotoxic and CD4+ expression as T helper cells. CD45RO and CD27 expression then defined memory cells that were subsequently analyzed for CD137 and IL-2 expression. [file Image_1.jpeg]
